# The Role of L-DOPA on Melanization and Mycelial Production in *Malassezia Furfur*

**DOI:** 10.1371/journal.pone.0063764

**Published:** 2013-06-07

**Authors:** Sirida Youngchim, Joshua D. Nosanchuk, Soraya Pornsuwan, Susumu Kajiwara, Nongnuch Vanittanakom

**Affiliations:** 1 Department of Microbiology, Faculty of Medicine, Chiang Mai University, Chiang Mai, Thailand; 2 Departments of Medicine (Infectious Diseases) and Microbiology/Immunology, Albert Einstein College of Medicine, Bronx, New York, United States of America; 3 Department of Physical Chemistry, Faculty of Science, Mahidol University, Bangkok, Thailand; 4 Department of Life Science, Graduate School of Bioscience and Biotechnology, Tokyo Institute of Technology, Yokohama, Japan; The University of Texas at San Antonio, United States of America

## Abstract

Melanins are synthesized by organisms of all biological kingdoms and comprise a heterogeneous class of natural pigments. Certain of these polymers have been implicated in the pathogenesis of several important human fungal pathogens. This study investigated whether the fungal skin pathogen *Malassezia furfur* produces melanin or melanin-like compounds. A melanin-binding monoclonal antibody (MAb) labelled *in vitro* cultivated yeast cells of *M. furfur*. In addition, melanization of *Malassezia* yeasts and hyphae was detected by anti-melanin MAb in scrapings from patients with pityriasis versicolor. Treatment of *Malassezia* yeasts with proteolytic enzymes, denaturant and concentrated hot acid yielded dark particles and electron spin resonance spectroscopy revealed that these particles contained a stable free radical compound, consistent with their identification as melanins. *Malassezia* yeasts required phenolic compounds, such as L-DOPA, in order to synthesize melanin. L-DOPA also triggered hyphal formation *in vitro* when combined with kojic acid, a tyrosinase inhibitor, in a dose-dependent manner. In this respect, L-DOPA is thought to be an essential substance that is linked to both melanization and yeast-mycelial transformation in *M. furfur*. In summary, *M. furfur* can produce melanin or melanin-like compounds *in vitro* and *in vivo*, and the DOPA melanin pathway is involved in cell wall melanization.

## Introduction

*Malassezia* species are lipophilic yeasts that are frequent components of the skin microflora of humans and most warm-blooded animals [1], [2]. Based on molecular data and lipid requirements, seven *Malassezia* species are now recognized [3], [4]. In humans, pityriasis versicolor (PV), known also as tinea versicolor, is a common superficial skin infection restricted to the stratum corneum that occurs when *Malassezia* yeast cells convert to a pathogenic mycelial form. *M. globosa* and *M. furfur* are significantly more common causes of PV than other species [5]. The disease is characterized by the presence of fine scaly patches or macules, which may be either hypo- or hyperpigmented, that are normally located on the upper parts of trunk, neck, and arms [5], [6]. PV is one of the most common pigmentary disorders worldwide, but it is more common in tropical climates, reaching as high as 50% in some tropical areas, which is attributed to the growth benefit achieved in the relative high temperature and humidity of these regions [5].

Since PV is one of the most common human skin infections, investigation into pathophysiological mechanisms underlying the disease can lead to a greater understanding of the causes, progression and outcomes of human *Malassezia* infections. However, to date, there is relatively little information on the virulence factors of *Malassezia* spp. The attributes of *Malassezia* yeasts implicated thus far in pathogenesis include lipolytic enzymes that injure host tissues and provide nutrients for the fungus [7]–[9], high lipid content of cell walls that protects *Malassezia* yeasts from phagocytosis [10] and downregulates the inflammatory immune response [11], [12], hyphae formation, and production of a tryptophan-dependent pigment that acts as protective barrier against the UVA and UVB spectrum [13], [14].

Melanins are biologically prominent macromolecules that are dark brown and black pigments formed by oxidative polymerization of phenolic compounds. Melanins can be classified into 3 typical types: eumelanin; formed by a complex polymerization process involving quinones and free radicals, phaeomelanin; derived from tyrosine and cysteine and allomelanins; formed from nitrogen free precursors [15]. Generally, two types of melanins, 1,8 dihydroxynaphthalene (DHN) and L-3,4- dihydroxyphenylalanine (DOPA) melanins are found in fungi, but most are synthesized from DHN-melanins. Melanins produced from acetate via the polyketide synthase pathway are normally black or brown and are referred to as DHN melanins. DOPA melanins are catalyzed by phenoloxidases (such as tyrosinases, laccases, or catecholases) and are called eumelanins [16].

They have been linked with virulence in an array of human pathogen fungi, such as *Cryptococcus neoformans* [17]–[19], *Aspergillus fumigatus* [20]–[24], *Histoplasma capsulatum* [25] and *Penicillium marneffei* [26]. Melanin has been shown to protect fungi in several ways. It functions as a beneficial physiologic redox buffer, protects pathogens against environmental stress such as UV radiation, temperature extremes, hydrolytic enzymes, metals or free radicals, and enhances resistance to antifungal drugs [19].

The study of melanization in *Malassezia* spp. has previously been investigated using the Masson-Fontana stain, which demonstrated the accumulation of black pigment on the cell wall of yeast cells both *in vitro* and during infection [27]. However, the Masson–Fontana silver stain is not specific for melanins, as evidenced by the fact that both melanized and non-pigmented *C. neoformans* cells are stained by this method [28]. In this report, we have confirmed the presence of melanins *in vitro*-melanized yeast cells of *M. furfur* by utilizing techniques developed to study and isolate melanins from other fungal pathogens. In addition, a melanin-specific monoclonal antibody (MAb) is used in the confirmation of melanization both *in vitro* and during human infection. We show that *M. furfur* produces DOPA-melanin. We also show that kojic acid, a tyrosinase inhibitor, can trigger the mycelial transformation in *M. furfur*, which is considered to be important in the pathogenesis of *Malassezia*.

## Materials and Methods

### Fungal Strain and Media

*M. furfur* NBRC 0656 was maintained by monthly subculture on Modified Dixon medium (mDixon; 1 liter of distilled water, 36 gm malt extract, 6 gm peptone, 20 gm ox bile (all obtained from Difco), 10 ml Tween 40, 2 ml glycerol, 2 ml olive oil (all obtained from Sigma) 0.05 gm chloramphenicol (Amresco), and 15.0 gm agar (Difco) with a pH 6.0 (modified from Guillot and colleague [29]). Unless otherwise specified, *M. furfur* was cultured on chemically defined minimal medium (MM) agar (15.0 mM glucose, 10.0 mM MgSO_4_, 29.4 mM KH_2_PO_4_, 13.0 mM glycine, 10.0 mM NH_4_Cl and 3.0 µM thiamine; pH 5.5) and supplemented with 0.1% (v/v) Tween 40, 0.1% (v/v) Tween 80 and 1.5% agar. For melanization studies, *M. furfur* was incubated at 30°C for 10–14 days in MM with or without L-3,4-dihydroxyphenylalanine (L-DOPA) (Sigma), at a concentration 1.0 mM. The *Malassezia* yeast cells were harvested by adding 3.0 ml sterile PBS to the culture plates and removed by gentle scraping with a loop. The yeast cells were collected by centrifugation at 4500 g for 20 min and the pellets were washed three times with sterile PBS.

### Growth of *M. furfur* in Media with Different Amino Acids as Nitrogen Sources

To study the effect of nitrogen sources, *M. furfur* yeasts were cultivated on basal medium (3.0% Tween 80 (Sigma, St Louis, MO, USA), 50.0 µg/ml of chloramphenicol (Amresco, Ohio, USA) and 2.0% agar (Difco) (modified from Mayser and colleagues [30]) with or without 15 mM of sterile filtrated L-tryptophan (Tryp; Sigma) or arginine (Arg; Sigma) as amino nitrogen source. To further investigate the effect of nitrogen sources, Tryp or Arg, at a concentration of 0.6% was added into mDixon medium instead of peptone as a nitrogen source. As a negative control, mDixon without peptone or amino acid was used. The plates were incubated at 30°C for 10–14 days.

### Isolation and Purification of Melanin Particles from *M. furfur*

The *Malassezia* yeasts were subjected to a melanin extraction protocol [31]. In brief, yeast cells were washed first with PBS and then with 1.0 M sorbitol and 0.1 M sodium citrate (pH 5.5). Lysing enzyme from *Trichoderma harzianum* (Sigma) was added at 10 mg/ml and incubated overnight at 30°C to generate protoplasts. The protoplasts were collected by centrifugation, washed three times with PBS, and incubated in 4.0 M guanidine thiocyanate (Sigma) overnight at room temperature. The resultant dark particles were collected by centrifugation, washed three times with PBS, and treated with 1.0 mg/ml Proteinase K (Roche) in reaction buffer [10.0 mM Tris, 1.0 mM CaCl_2_ and 0.5% (w/v) SDS, pH 7.8] and incubated at 37°C. The resultant debris was washed three times with PBS and boiled in 6.0 M HCl for 1.5 hours. After treatment by boiling in acid, melanin particles were collected by filtration through Whatman paper no. 1 and were washed extensively with distilled water. Melanin particles were then dialyzed against distilled water for 10 days until the acid was completely removed, whereupon they were lyophilized.

### Electron Spin Resonance (ESR) Spectroscopy Analyses

ESR spectroscopy is a technique that directly detects the signal from a free electron spin or free radical. Due to the abundance of stable free radicals in melanin pigments, ESR is used as a tool to indicate the presence of melanin in the samples by using a Gunn diode as a microwave source [32]. The ESR signal of melanin in derivative mode shows a typical single peak located in the middle of the spectrum at approximately 3500 Gauss. We used this method with a total of 2 g freeze-dried material in each case (analysis was carried out in silica cuvettes). *Aspergillus fumigatus* and *P. marneffei* melanins were used as positive controls [24], [26].

### Immunofluorescence Analyses of Melanin in *M. furfur in vitro*

Suspensions of yeast cells from *M. furfur* grown on mDixon or MM with L-DOPA were air-dried on poly-L-lysine slides, and then incubated with Superblock Blocking Buffer in PBS (Pierce) for 2 h at 37°C or overnight at 4°C to block non-specific binding. The slides were washed three times with PBS, then incubated for 1.5 h at 37°C with 10 mg/ml of anti-melanin MAb 8D6 (against melanin of *A. fumigatus*; [24]) made up in Superblock blocking buffer in PBS. After washing three times with PBS, slides were incubated with a 1∶100 dilution of fluorescein-isothiocyanate-conjugated goat anti-mouse IgM (Jackson Immunoresearch Laboratories) for 1.5 h at 37°C. The slides were washed three times with PBS to eliminate unbound antibody and then mounted in glycerol-PBS (1∶1 v/v). Coverslips were applied and slides were examined using a Nikon fluorescence microscope Eclipse 50i. For a negative control, conjugated goat–anti mouse IgM (Jackson) alone without primary MAb was included in the experiments. In addition, *Saccharomyces cerevisiae* MMCM 5211 was used as negative control. Images were captured with Nikon DS Fi1.

### Phenoloxidase Plate Assay

A semi-qualitative laccase assay evaluating the oxidation of L-DOPA in *M. furfur* was modified from Srinivasan et al. [33] and Crowe and Olsson [34]. For the laccase plate assay, 15 ml of melting sterile MM agar with 1.0 mM L-DOPA was placed in a sterile petri dish (90 by 15 mm) containing three sterile cups (6 mm in diameter). The cups were removed after the agar was solidified. Two hundred µls of a yeast cell suspension (adjusted to McFarland No.5) in sterile PBS was inoculated into each well and the plates were incubated at 28°C for 7 to 10 days. *C. neoformans* H99 and *S. cerevisiae* MMCM 5211 were used as positive and negative controls, respectively. All plates were inspected daily for pigment production. The development of dark black color around the wells was considered as a positive reaction for laccase activity.

### Effect of Kojic Acid on Melanization of *M. furfur*

Kojic acid, an inhibitor of 3,4-dihydroxy phenylalanine [DOPA] melanin synthesis, was used to identify the type of melanin in *M. furfur*. *M. furfur* was cultured with kojic acid at different concentrations, 0, 600, 800, 1000, 1200 and 1500 µg/ml, in defined MM with or without 1.0 mM L-DOPA supplemented with 0.1% Tween 40, 0.1% Tween 80 at 30°C in for 12–14 days in the dark and examined for pigment production. After incubation, the *Malassezia* yeasts were stained with melanin-binding MAb 8D6, then harvested and subjected to melanin extraction as described above in 2.3 to further analyze recovered debris by ESR.

### Induction the Mycelial Phase of *M. furfur in vitro* by Using L-DOPA and Kojic Acid

In order to investigate the optimal condition for inducing mycelial production, the *Malassezia* cells were incubated in semi-solid (0.5% agar) culture media of MM consisting of lipid supplements and 1.0 mM L-DOPA with various concentrations of kojic acid. To demonstrate the requirement for L-DOPA, agar containing dopamine or L-tyrosine with kojic acid was also tested. The cultures were incubated at 30°C for 5–7 days in microaerophilic conditions using an anaerobic plastic jar with a microaerobic atmosphere generating system (Pack MicroAero, Mitsubishi Gas Chemical Co. Inc), and then viewed under light microscopy (magnification×1000) to determine the percentages of hyphal production. For each condition, a total of 1,000 fungal cells were counted to determine the percentage of filamentation. In addition, *M. furfur* NBRC 10987, CBS 6000, 6001, 6046, 6094 and 7966 (patient isolates kindly received from Dr. Teun Boekhout, Centraal bureau voor Schimmelcultures (CBS-KNAW), Yeast Research Group, Utrecht, Netherlands) were examined for their capacity to form filaments under these conditions. Also, two isolates of *M. furfur* from patients without skin disease, H_1_ and H_2_, were included in this study.

### Detection Melanization of Pityriasis Versicolor from Infected Skin

Skin scale specimens from patients with hypopigmented or hyperpigmented PV lesions were obtained from the Dermatological Clinic, Maharaj Nakorn Chiang Mai Hospital, Chiang Mai University. The skin scales utilized in the experiments were obtained in the course of routine care of patients with dermatological conditions. Written consent was obtained from all patients undergoing dermatological scrapings, and the consent included that the tissues may be utilized for experimental purposes, with the exception of human genetic studies, without further consent. The procedure was approved by the ethics committee of Chiang Mai University. Direct examination of skin was performed and the presence of *Malassezia* was confirmed by culturing on mDixon agar incubated at 30°C for 2 weeks. The *Malassezia* yeasts were identified to species level using polymerase chain reaction (PCR) as described [35]. To examine *Malassezia* melanization *in vivo*, the skin was digested with 1.0 mg/ml of Proteinase K (Roche) at 37°C for 2 h, and washed three times with PBS (modified from Youngchim et al. [36]). The skin was then probed with the melanin-binding MAb 8D6 and FITC–conjugated goat anti-mouse IgM as described above. Negative controls without primary MAb were included.

### Statistical Analysis

The percentages of filament production in *M. furfur* cultured in MM with L-DOPA and kojic acid at different concentrations were compared to control, *M. furfur* in MM or MM with L-DOPA by two tailed, unpaired Student’s t-test using Prism 4 software (GraphPad). A *P*-value ≤0.05 was considered significant.

## Results

### Melanization of *M. furfur* Yeast Cells *in vitro*

**Figure 1:**
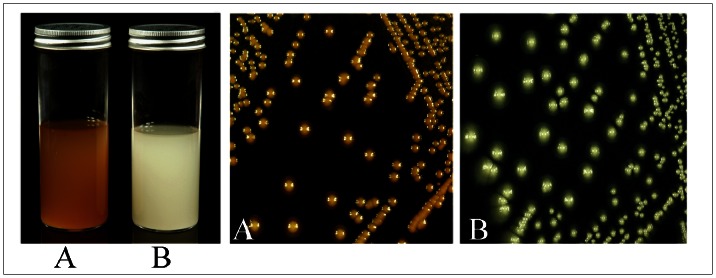
Growth of *M. furfur* NBRC 0656 in minimal media with (A) and without L-DOPA (B).

*M. furfur* NRBC 0656 was cultured on MM with or without L-DOPA for 7–10 days at 30°C. All cultures were observed daily for pigment production. Growth of *Malassezia* required the inclusion of a lipid source in the culture medium. After a period of 7 days, the *Malassezia* colonies grown on MM supplemented with L-DOPA displayed a dark brown pigment (Figure 1A), and the intensity of the dark color increased with continued incubation. Treatment of these pigmented cells by the melanin extraction protocol resulted in the collection of aggregated dark particles. In contrast, *M. furfur* yeast cells grown in minimal medium without L-DOPA were macroscopically non-pigmented (Figure 1B), and the non-pigmented cells were completely solubilized by subjecting them to the melanin extraction protocol.

### ESR Spectroscopy of *M. furfur* Melanin

**Figure 2:**
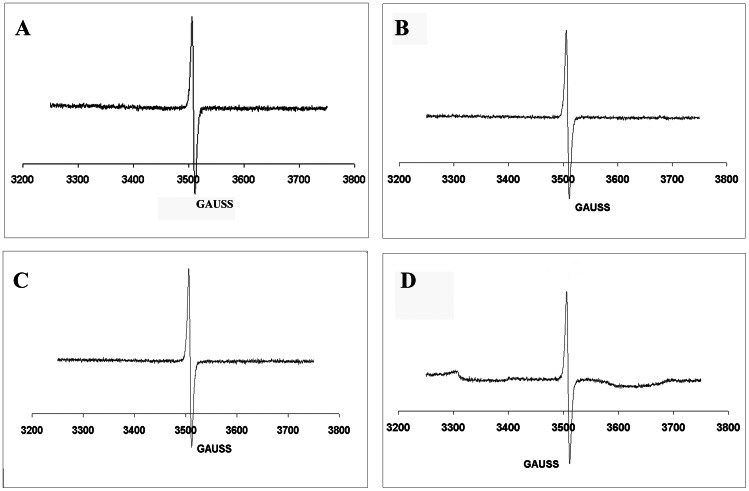
ESR spectroscopy of melanin particles extracted from *M. furfur* NBRC 0656 grown on defined minimal medium with L-DOPA (A), mDixon medium (B), basal medium with L-Tryp (C) and basal medium with Arg (D) at 30°C for 7–10 days.

ESR spectroscopy of the melanin-like particles collected from *M. furfur* NBRC 0656 grown on minimal medium with L-DOPA produced signals indicative of the presence of a stable free-radical population consistent with the pigment being identified as melanins (Figure 2A) [32]. The spectrum was indistinguishable to the signals produced with melanins of other pathogenic fungi such as *C. neoformans* [31], *H. capsulatum* [25], *A. fumigatus* [24] and *P. marneffei* [26]. The black particles isolated from *M*. *furfur* grown on mDixon medium (Figure 2B) and basal media with L-Tryp or Arg (Figure 2C and D, respectively) also produced ESR signals consistent with melanin. In contrast, *M. furfur* cultured on MM without L-DOPA was completely dissolved when subjected to melanin extraction protocol.

### Immunofluorescence (IF) Reactivity of the Anti-melanin MAb 8D6 to *M. furfur* Yeast Cells before and after Melanin Extraction

**Figure 3:**
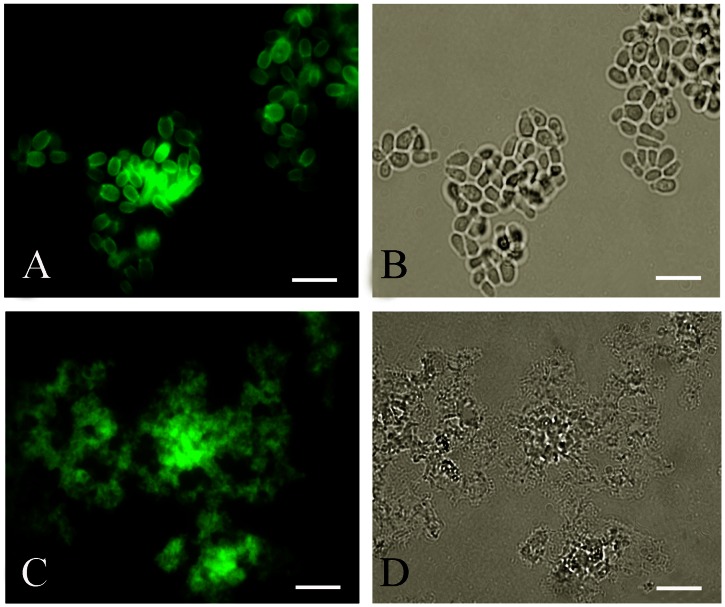
Corresponding immunofluorescence and bright-field microscopy images demonstrating the labelling of yeast cells of *M. furfur* NBRC 0656 (A,B) and after (C,D) melanin extraction by the melanin-binding MAb 8D6. Bars represent 5 µm.

*M. furfur* yeast cells grown in both mDixon medium and MM with L-DOPA were reactive with melanin-specific MAb 8D6, showing a pronounced fluorescent reactivity within the cell wall (Figure 3A,B). In contrast, *M. furfur* grown on MM without L-DOPA was not labelled by MAb 8D6. After the melanin extraction procedure on the yeast cells grown in mDixon medium, the resultant particles in both isolates of *Malassezia* yeast cells retained reactivity to MAb 8D6 (Figure 3C,D). There was no reactivity with *Malassezia* yeast cells or melanin particles when the secondary MAb was used alone (data not shown). *S. cerevisiae* was also negative for anti-melanin MAb 8D6.

### Phenoloxidase Plate Assay

**Figure 4:**
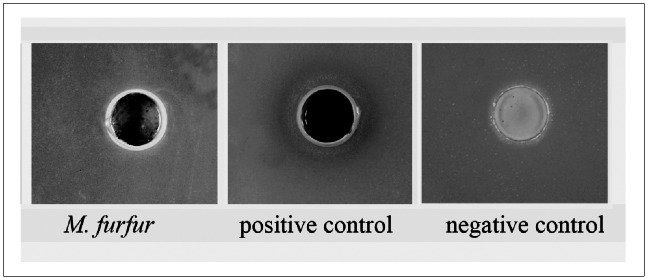
Plate assay for demonstration of phenoloxidase activities for 10 days. *M. furfur* NBRC 0656 was inoculated into wells in laccase agar plate containing 1 mM of L-DOPA as the substrate. *C. neoformans* H99 and *S. cerevisiae* MMCM 5211 were used as positive and negative controls, respectively. The development of an intense black color around the well indicated a positive test for phenoloxidase activity.

The phenoloxidase plate assay was based on the ability of an organism to oxidize L-DOPA incorporated into the media, resulting in black color production around the *Malassezia* cell suspensions. The results of the phenoloxidase plate assay (Figure 4) demonstrated the presence of laccase activity in cell suspensions of *M. furfur* NBRC 0656 and *C. neoformans* H99 (positive control), but no reactivity occurred with *S. cerevisiae* (negative control).

### Effect of Kojic Acid on Melanization of *M. furfur* NRBC 0656

**Figure 5:**
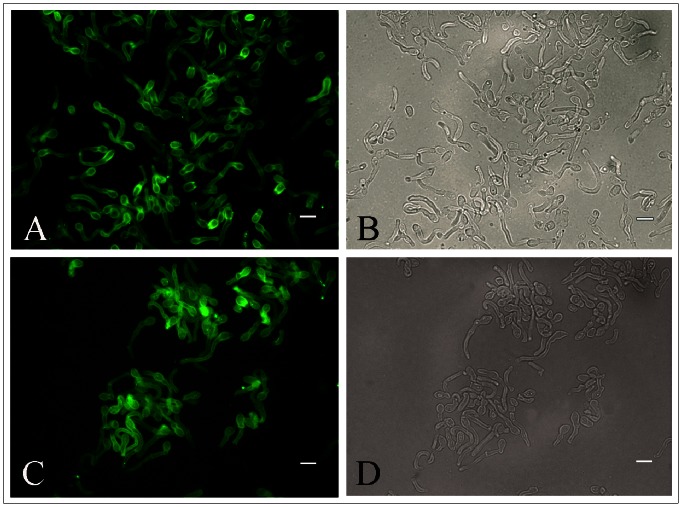
Corresponding immunofluorescence and bright-field microscopy images demonstrating the labelling of yeast cells of *M. furfur* NBRC 0656 (A,B) and CBS 7019 (C,D) grown on MM with 1 mM L-DOPA and 1500 µg/ml kojic acid by the melanin-binding MAb 8D6. Bars represent 5 µm.

Kojic acid blocked L-DOPA melanization in *M. furfur* (Figure S1). Whereas yeast on MM with 1.0 mM L-DOPA developed a dark brown color (Figure S1B), as little as 100 µg/ml of kojic acid reduced the pigmentation of *M. furfur* (Figure S1C). At 600 µg/ml of kojic acid the colonies appeared as a light yellow (Figure S1F) and at 1000 µg/ml (Figure S1H) the colonies and the plates were equivalent in appearance to yeast cultivated on MM without L-DOPA. Hence, kojic acid significantly reduced melanization in *M*. *furfur* supporting the involvement of the DOPA pathway in melanin synthesis. However, although kojic acid was able to block grossly observable melanization in *M. furfur,* these organisms were still reactive to melanin-binding MAb (Figure 5). In addition, the black particles were obtained when the mycelial cells of *M. furfur* cultured in 1 mM L-DOPA and 1500 µg/ml of kojic acid were treated with the melanin extraction protocol (data not shown).

### Induction the Mycelial Phase of *M. furfur* with L-DOPA and Kojic Acid

**Figure 6:**
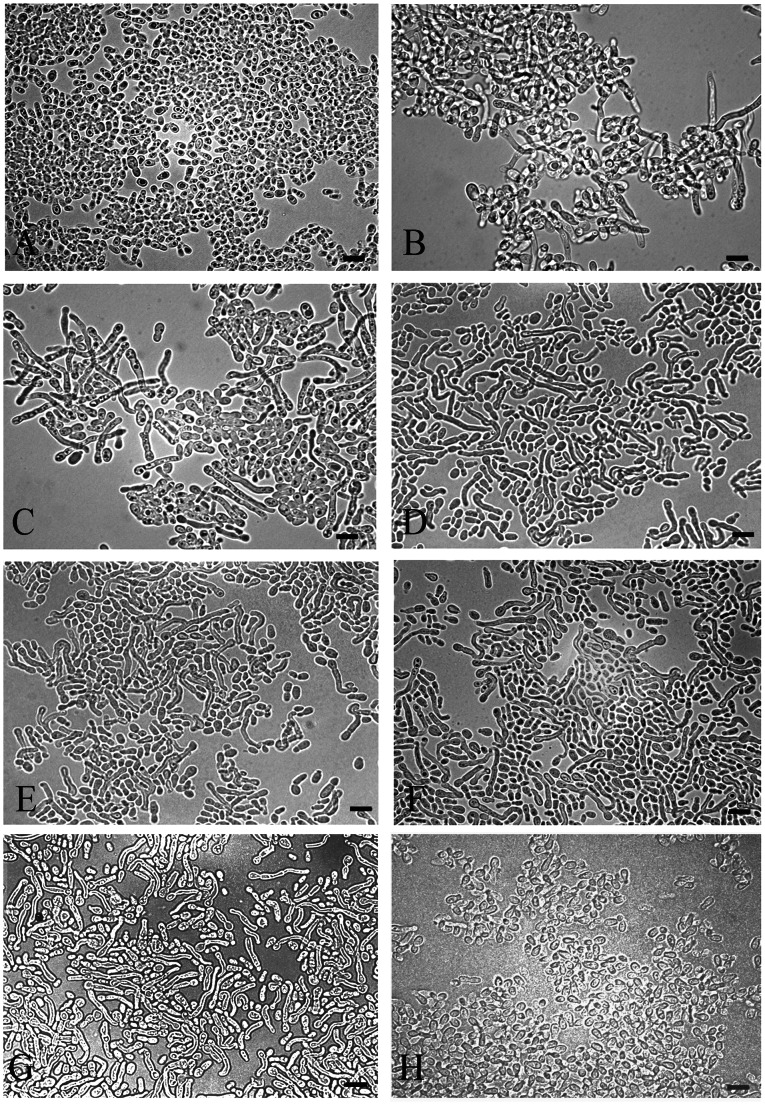
The effect of L-DOPA and kojic acid on morphology of *M. furfur* NBRC 0656 when culturing in MM (A), MM with 1 mM L-DOPA (B), MM with 1 mM L-DOPA and kojic acid at 600 (C), 800 (D), 1,000 (E) 1,200 (F) 1,500 (G) µg/ml, and MM with 1,000 µg/ml kojic acid (H). Bars represent 5 µm.

**Figure 7:**
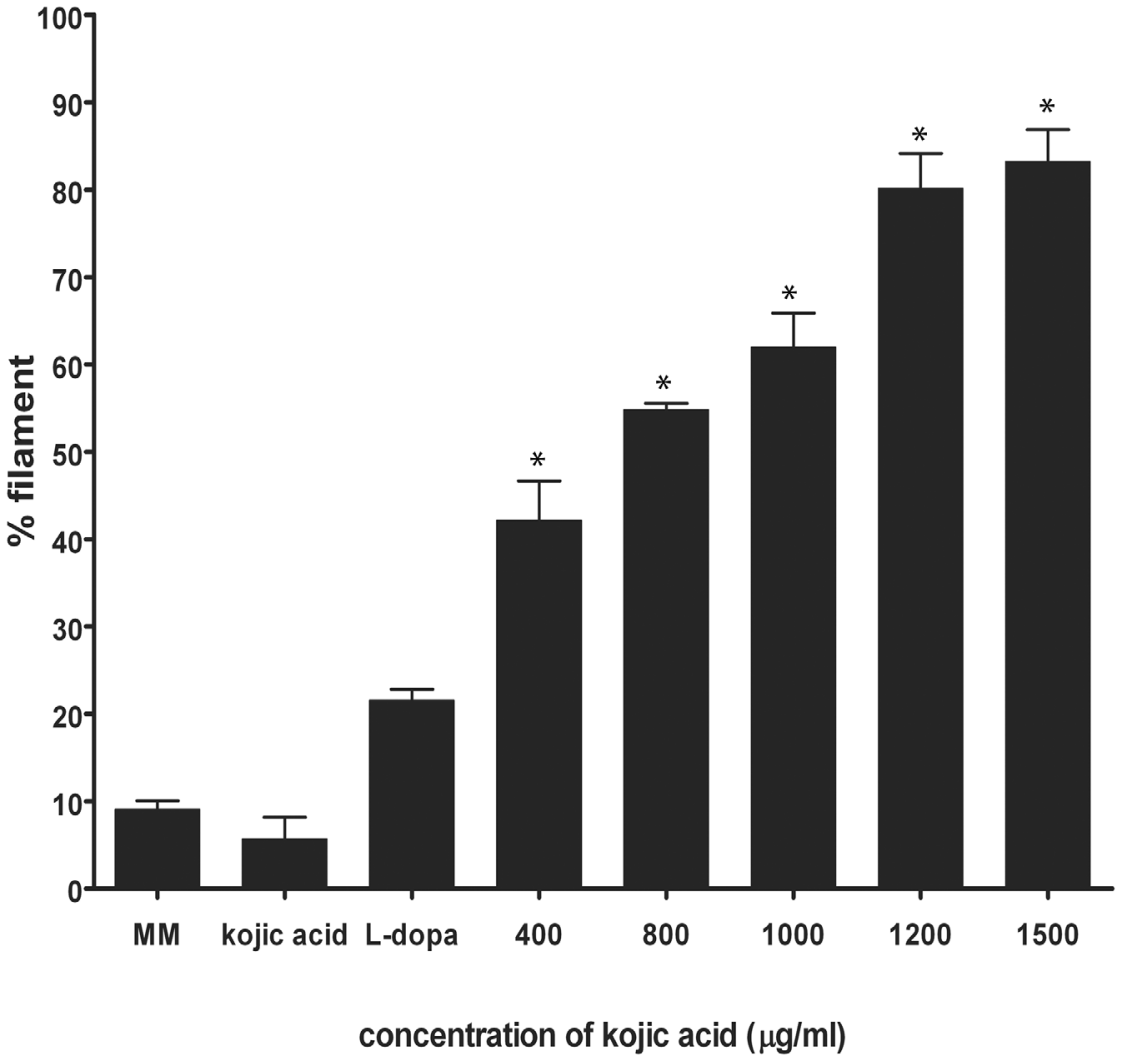
Effect of various concentrations of kojic acid on filament formation in *M. furfur* NBRC 0656 in microaerobic condition. Data are means±SD (standard deviation of the means) based on at least three experiments. Statistically significant difference (two-tailed unpaired Student’s t-test) compared to control, MM and MM with L-DOPA, *p≤0.05.

**Figure 8:**
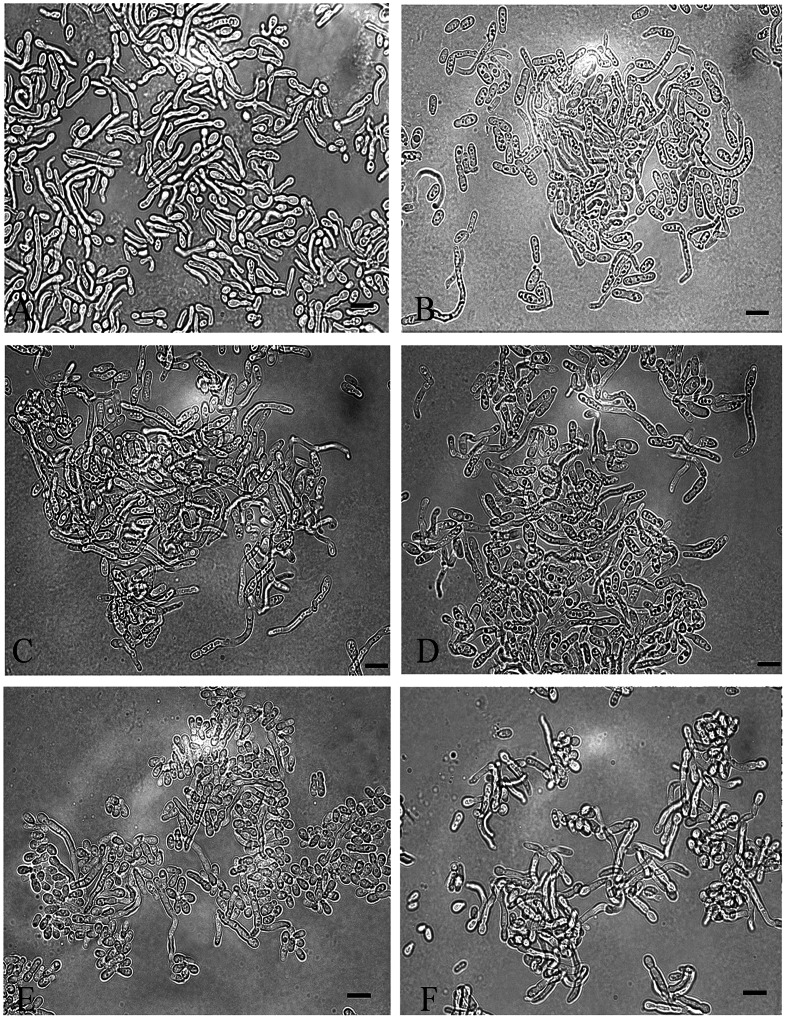
The mycelial productions of the various isolates in *M. furfur*. *M. furfur* CBS 6046 (A), CBS 6000 (B), CBS 6001 (C), CBS 7019 (D) and two isolates from healthy person (E, F) were cultured on MM with 1 mM L-DOPA and kojic acid in 0.5% agar in microaerophilic environment at 30°C for 5–7 days. The concentrations of kojic acid were 1500 µg/ml for standard isolates (A-D) and 500 µg/ml for healthy isolates (E,F). Bars represent 5 µm.

In addition to the inhibitory effect of kojic acid on melanization of *Malassezia*, we observed that the addition of kojic acid and L-DOPA into MM induced the conversion of yeast cells to short filament (mycelial phase) forms (Figure 6). Although L-DOPA alone induced hyphal transformation, the percentage of hyphae increased in a dose dependent manner with the addition of kojic acid (Figure 6B–G and 7). Notably, kojic acid was incapable of inducing filamentation in *Malassezia* yeasts in the absence of L-DOPA (Figure 6H). The short filament transformations were also seen in other isolates of *M*. *furfur* including CBS 6000, 6001, 7019 and two additional strains from healthy individual incubated with L-DOPA and 1500 µg/ml of kojic acid (Figure 8). In contrast, neither dopamine nor L-tyrosine was effective in stimulating mycelial production in *M. furfur* when co-cultured with kojic acid (data not shown).

### Detection of Melanization in *Malassezia* Infected Skin

**Figure 9:**
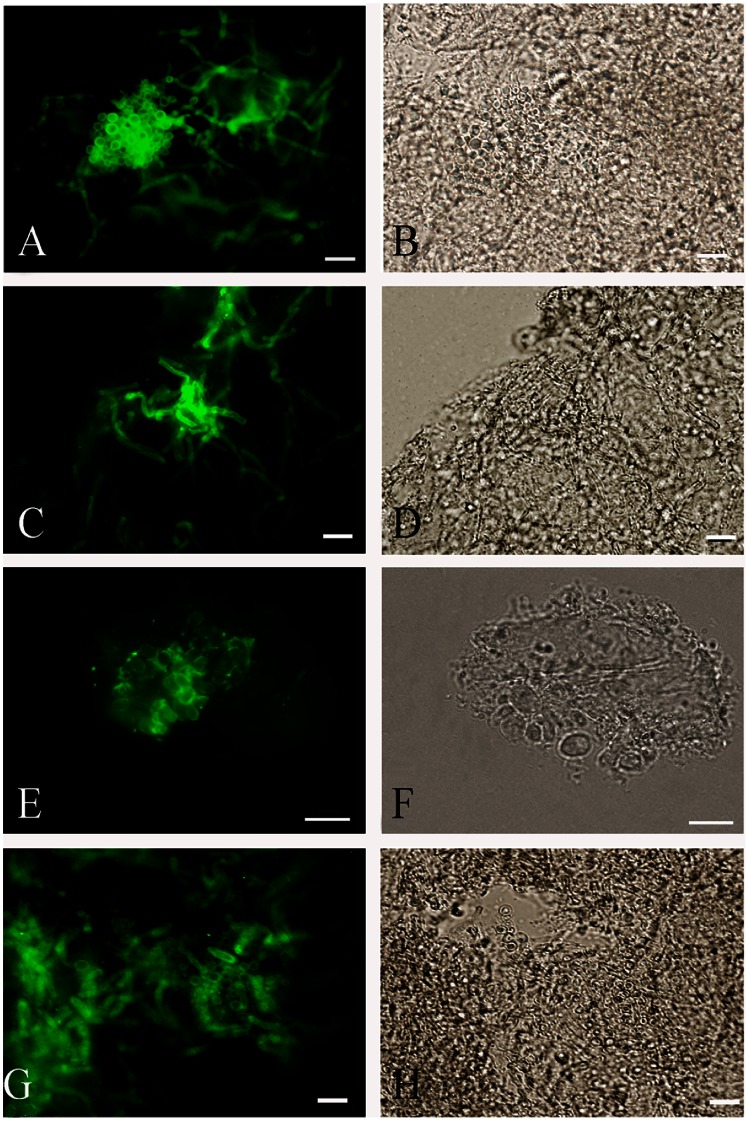
Corresponding immunofluorescence (A,C,E,G) and bright-field microscopy (B,D,F,H) images demonstrating the labelling of blastoconidia and short filaments from skin scrapings of pityriasis versicolor by the melanin-binding MAb 8D6. **Bars represent 5 µm.**

Skin scrapings from three patients with PV were stained by KOH preparation to confirm the presence of budding yeast cells and short filaments in samples. *M. globosa* was identified in two samples of PV with hyper- and hypopigmented lesions, while *M. furfur* was isolated from a hypopigmented lesion. The round budding yeast cells and short filaments in skin scale specimens from either hyperpigmented or hypopigmented PV lesions were strongly reactive with melanin-binding MAb 8D6 (Figure 9). No reactivity was observed when fluorescein isothiocyanate (FITC)-conjugated goat anti-mouse IgM was incubated without the primary MAb.

## Discussion

Melanins have been recognized as an important virulence determinant in several important human fungal pathogens, since there is extensive evidence that the pigment supports survival in a host and promotes the capacity of the organisms to cause diseases [37]. In this study, we have confirmed experimentally that melanin is synthesized by *Malassezia* yeasts, which parallels the data of Gaitanis et al. [27]. Our evidence supporting the melanization of *Malassezia* yeasts is as follows: (i) ESR spectroscopy analysis of black particles indicated the presence of a stable free radical compound consistent with melanin, (ii) reactivity of a melanin-binding MAb to the cell wall of *Malassezia* yeasts grown *in vitro* and with the pigmented particles derived from these cells, (iii) demonstration of the presence of a phenoloxidase, and (iv) reactivity of a melanin-binding MAb to the yeast cells and short hyphae in scrapings from *Malassezia* infected skin. The ESR results represent the first biophysical evidence of the production of melanin in *M. furfur* and the enzymatic plate assay the first chemical evidence for a phenoloxidase in *Malassezia*. In addition, the melanin-binding MAb 8D6 raised against DHN melanin from *A. fumigatus* was applied to detect melanization in *M. furfur* both *in vitro* and *in vivo*. The MAb 8D6 has been previously showed to react with DHN and DOPA melanins from other fungi [24]. Clearly, DHN melanin must share some epitopes with DOPA melanin, which was also shown in other studies [18], [38], [39]. Together these observations provide solid evidence that *Malassezia* can synthesize melanin or melanin-like compound *in vitro* and *in vivo*. In our study, *Malassezia* yeasts and hyphae in both hyper- and hypopigmented PV skin lesions were strongly reactive with melanin-specific MAb indicating that melanization occurs *in vivo* in both forms of PV.

Interestingly, the study by Mayser and Pape [13] demonstrated that synthesis of pigments and fluorochromes by *M. furfur* provided potent protection from UVA and UVB range radiation when compared with the survival of non-pigmented cells. The documentation of melanin production by *M. furfur* supports that the polymer is involved in the protection against UV irradiation, given the data on the radioprotection of fungal melanins [37], [40]. Interestingly, the radioprotective efficacy of fungal melanins is a function of the chemical composition and spatial arrangement of the polymers [41].

*Malassezia* species are dimorphic fungi capable of yeast-mycelial change. During *Malassezia* infection, round or oval yeast cells switch to mycelia forms [42]. Thus, the production of hyphae is considered to be associated with virulence. To investigate the pathogenicity and antimycotic susceptibility of *Malassezia*, investigators have sought to induce the growth of the mycelial form *in vitro*, but prior attempts to produce media for hyphal transformation have been unsuccessful. In experiments using human stratum corneum, investigators have only able to obtained 24% of hyphae in *Pityrosporum ovale* (*M. furfur*) [43]. It has been suggested that one or several substances in the stratum corneum are essential to activate the production of hyphae. In our experiments, *M. furfur* was completely transformed to hyphae when adding L-DOPA and kojic acid in MM medium with lipids. This finding raises the intriguing possibility that *Malassezia* yeasts are able to produce a tyrosinase inhibitor to acquire L-DOPA in the host environment. This assumption is supported by a previous study of Nazzaro-Porro and Passi [44] who found tyrosinase inhibitors in the cultures of *Pityrosporum orbiculare* (*M. furfur*) supplemental with oleic acid or vaccenic acid. The tyrosinase inhibitors secreted by *Malassezia* spp probably suppress melanin formation resulting in depigmentation in human skin but without effect on melanization of *Malassezia*. Hence, this inhibitor may have an inhibitory effect that differs from kojic acid which inhibits tyrosinase as well as prevents the conversion of the *o*-quinones of L-DOPA [45]. Due to the basal culture medium in our studies, MM (starvation medium) with lipids is required for mycelial induction of *Malassezia in vitro,* which is similar to the study of Dorn and Roehnert [46].

Apart from an inhibitory effect of tyrosinase, kojic acid appears to affect the morphology of *Malassezia* yeasts. Kojic acid possesses many activities on diverse fungi including antifungal activities against *Candida albicans*, *C. neoformans*, and *Trichophyton rubrum* [47], and can also affect aflatoxin production in *A. flavus* [48]. It appears that this inhibitor could induce the changes in *Malassezia* yeasts that occur on healthy skin, causing them to develop filaments and convert to the parasitic form.

In our study, phenoloxidase was detected in *M. furfur* which implied that DOPA melanin formed in this organism, similar to the study by Gaitanis et al. [27]. In addition to our finding that L-DOPA can impact mycelial synthesis, L-DOPA is an essential substrate for melanin production in *Malassezia* spp. Therefore, *Malassezia* conversion in* vivo* depends on the presence of L-DOPA in the epidermis. There have been two studies supporting the occurrence of L-DOPA in the epidermis. First, L-DOPA has been detected in the skin of pigmented animals by gas chromatography [49]. Second, Langerhans cells have a L-DOPA transport mechanism [50], suggesting that the compound is presented and involved in biological responses in local tissues.

The filament production in *Pityrosporum* (*Malaassezia*) has been shown to be more prominent in a microaerophilic environment [51]. Accordingly, our results are in agreement with previous studies, which can provide an explanation for the survival of *Malassezia* spp in the deepest parts of hair follicles [52] or the stratum corneum [53], [54], growing both in yeast and mycelial forms [52].

In conclusion, our results demonstrated that *M. furfur* synthesizes melanin or a melanin-like pigment when grown *in vitro* and *in vivo*. L-DOPA and kojic acid are thought to play a role in triggering the hyphal synthesis in *M. furfur* when incubated in a microaerophilic environment. Further studies should be investigated in other species of *Malassezia* yeasts isolated from healthy and PV skin lesions in both hyper-and hypopigmented lesions, in particular for species identification and their capacity to form hyphae *in vitro*. In addition, genetic manipulation strategies have recently been developed for *Malassezia* spp and these will be instrumental in prospective studies aimed at investigating the genes responsible for melanin formation and tyrosinase inhibitors.

## Supporting Information

Figure S1 **The colony morphology of** ***M. furfur*** **NBRC 0656 on MM (A), MM with 1 mM L-DOPA (B), MM with 1 mM L-DOPA and various concentrations of kojic acid; 100 (C), 200 (D), 400 (E), 600 (F), 800 (G) and 1000 (H) µg/ml.**
(TIF)
