# Supplementary figures and images for: The Role of L-DOPA on Melanization and Mycelial Production in Malassezia Furfur
Source: PLoS One. 2013 Jun 7;8(6):e63764. doi: 10.1371/journal.pone.0063764 (PMC3676409; doi:10.1371/journal.pone.0063764)

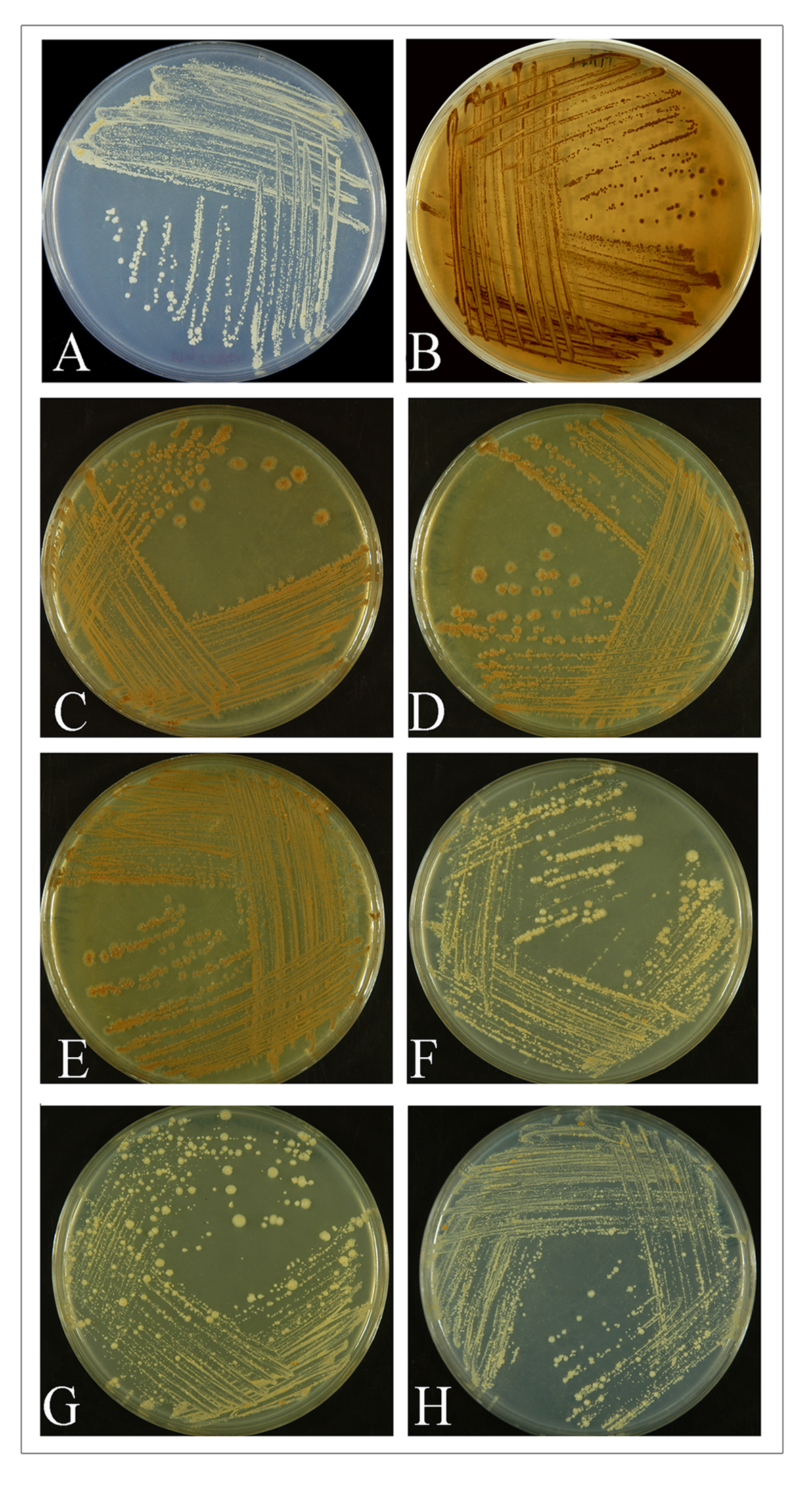

Supplement: Figure S1 — The colony morphology of M. furfur NBRC 0656 on MM (A), MM with 1 mM L-DOPA (B), MM with 1 mM L-DOPA and various concentrations of kojic acid; 100 (C), 200 (D), 400 (E), 600 (F), 800 (G) and 1000 (H) µg/ml. (TIF) [file pone.0063764.s001.tif]
